# Supplementary material for: Characterization of Early and Late Damage in a Mouse Model of Pelvic Radiation Disease
Source: Int J Mol Sci. 2023 May 15;24(10):8800. doi: 10.3390/ijms24108800 (PMC10218716; doi:10.3390/ijms24108800)
Supplement: Supplementary file 1 [file ijms-24-08800-s001.zip › Table S1.pdf]

Table S1. List of primers used for Real-Time PCR

	Primer Forward 5'-3'	Primer Reverse 5'-3'
**mLGR5**	ACCCGCCAGTCTCCTACATC	GCATCTAGGCGCAGGGATTG
**mAscl2**	CTACTCGTCGGAGGAAAG	CTAGACAGCATGGGTAAG
**mSmoc2**	CAGGTCCAGTGTCACAGCTACAC	GGTCTTGTTCTGCCGACTCTTAAC
**mLrig1**	AAGGGAACTCAACTTGGCGAG	ACGTGAGGCCTTCAATCAGC
**mTert**	GCAGGTGAACAGCCTCCAGACAG	TCCTAACACGCTGGTCAAAGGGAAGC
**mEphb2**	TGTGGAGCTATGGCATCGT	TGGGCGGAGGTAGTCTGTAG
**mBMI1**	CCAATGAAGACCGAGGAGAA	TTTCCGATCCAATCTGCTCT
**mZNF**	GAAGCACATGATTATCGAGCA	AATCAGTGATGGGCTGTTCA
**mProm1**	TGGTCCAGCCGAATGACTTC	TCCGATCTCCTTGGAATCAACT
**mIL-1β**	CAGGCAGTATCACTCATTG	CGTTGCTTGGTTCTCCTTGT
**mIL-6**	CAAGTCGGAGGCTTAATTACACATG	TGCCATTGCACAACTCTTTTCT
**mTNFα**	CAGACCCTCACACTCAGATCATCTT	CCACTTGGTGGTTTGCTACGA
**miNOS**	GGTGAAGGGACTGAGCTGTTAGA	GTTCTCCGTTCTCTTGCAGTTGA
**mCOX-2**	TGCACTATGGTTACAAAAGCTGG	TCAGGAAGCTCCTTATTTCCCTT
**mBcl-2**	GCATGCGACCTCTGTTTGATT	CCAGGCTGAGCAGGGTCTT
**mBAX**	GTGAGCGGCTGCTTGTCT	GGTCCCGAAGTAGGAGAGGA
**mp21**	CGAGAACGGTGGAACTTTGAC	CAGGGCTCAGGTAGACCTTG
**mp19**	TGCGCTCTGGCTTTCGTGAA	GAGCAGAAGAGCTGCTACG
**mZO-1**	CGGTCCTCTGAGCCTGTAAG	GGATCTACATGCGACGACAA
**mALPI**	TAACTCACCTCATGGGCCTCT	GGTCTACTGAGGGGTTTCGG
**mGAPDH**	AACTTTGGCATTGTGGAAGG	CACATTGGGGGTAGGAACAC
