# Supplementary figures and images for: Characterization of Early and Late Damage in a Mouse Model of Pelvic Radiation Disease
Source: Int J Mol Sci. 2023 May 15;24(10):8800. doi: 10.3390/ijms24108800 (PMC10218716; doi:10.3390/ijms24108800)

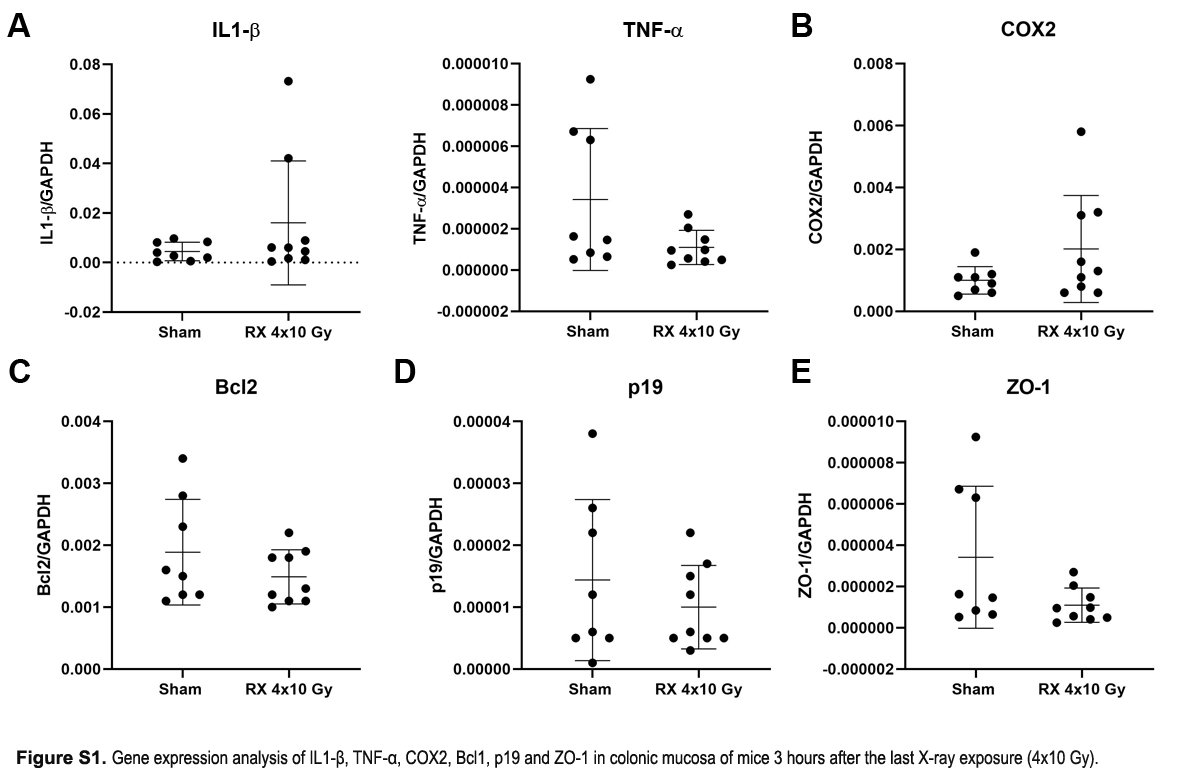

Supplement: Supplementary file 1 [file ijms-24-08800-s001.zip › FigureS1.tif]

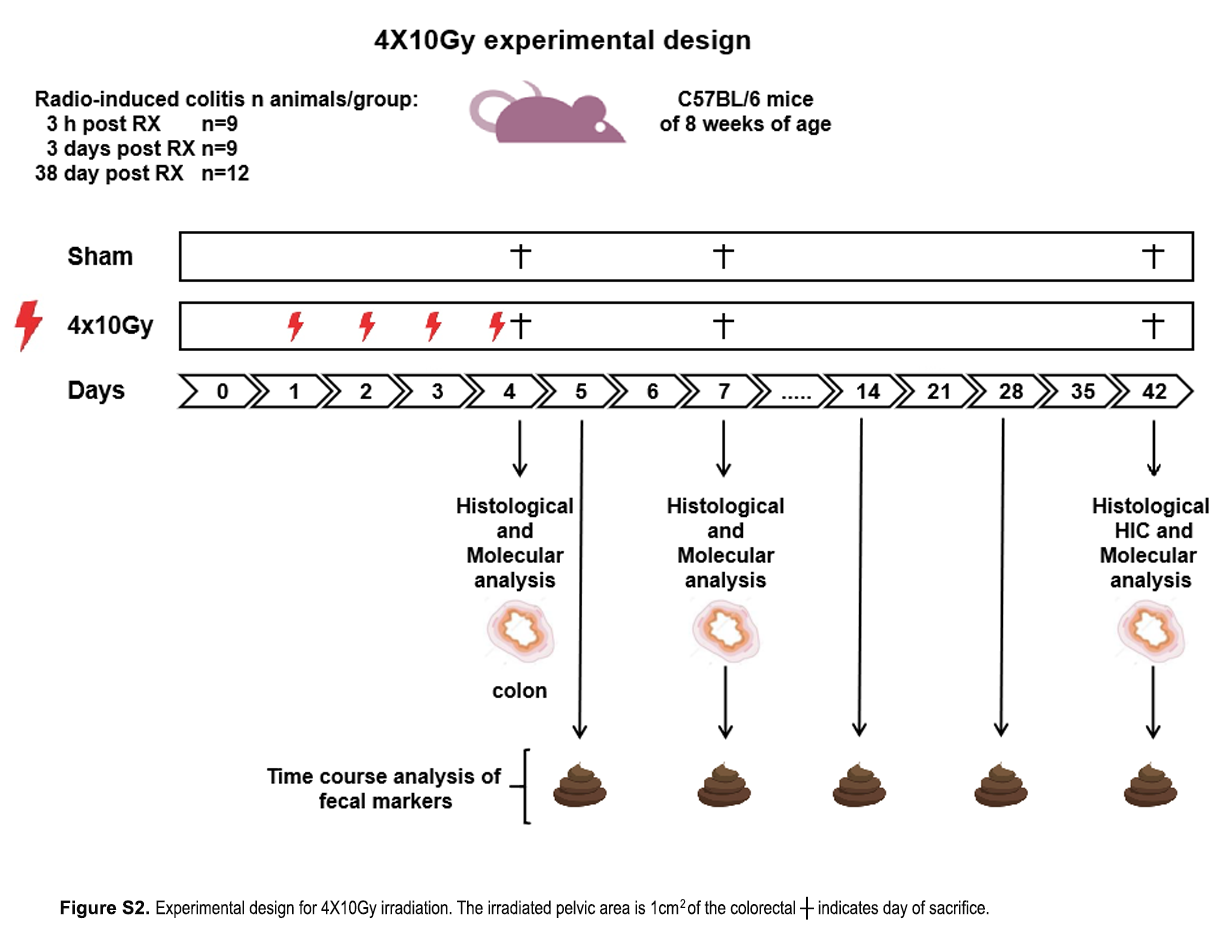

Supplement: Supplementary file 1 [file ijms-24-08800-s001.zip › FigureS2.tif]

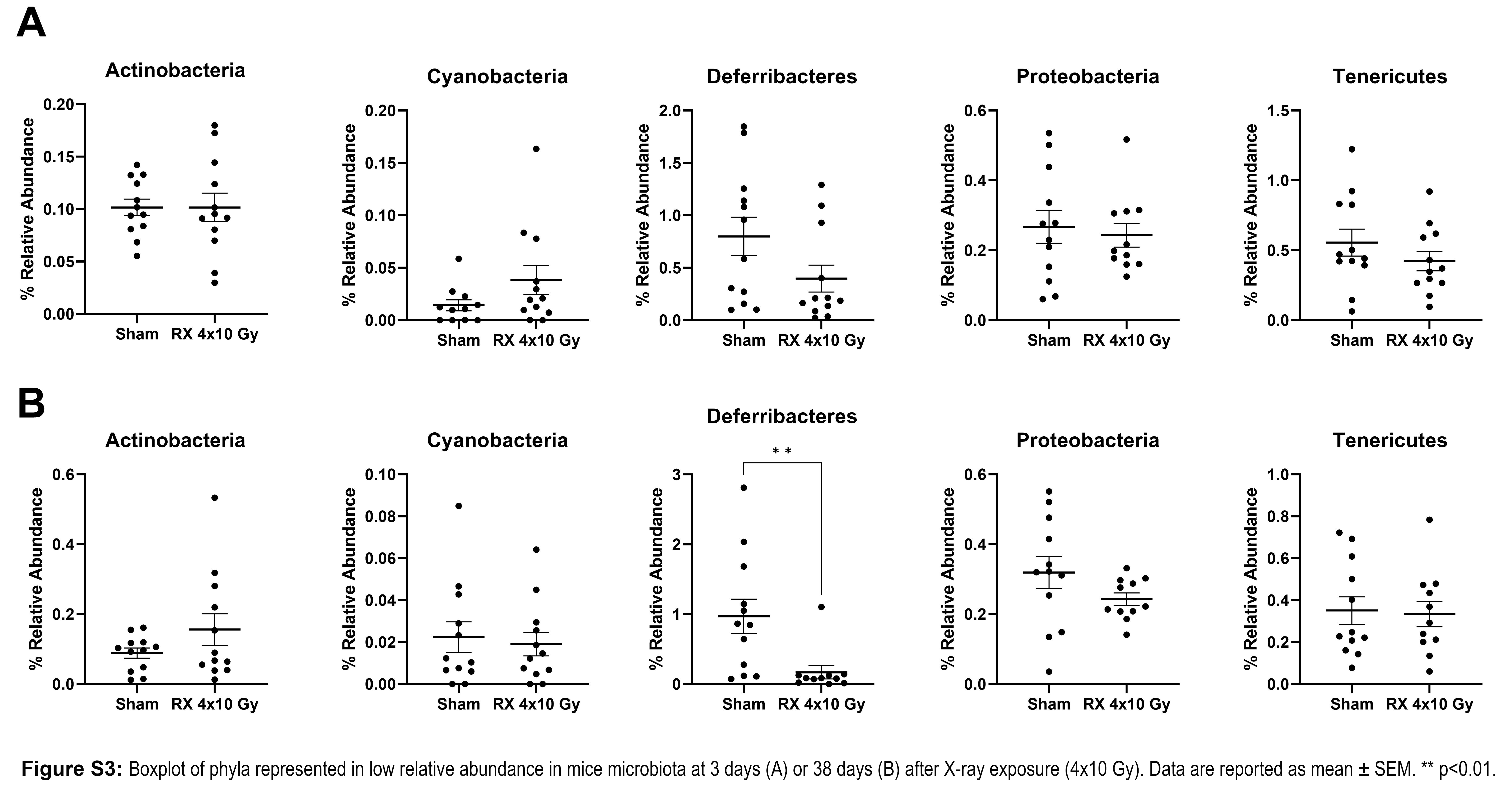

Supplement: Supplementary file 1 [file ijms-24-08800-s001.zip › FigureS3.tif]
